# Managing the airway catastrophe: longitudinal simulation-based curriculum to teach airway management

**DOI:** 10.1186/s40463-019-0332-0

**Published:** 2019-02-19

**Authors:** Lily H. P. Nguyen, Ilana Bank, Rachel Fisher, Marco Mascarella, Meredith Young

**Affiliations:** 1 Department of Otolaryngology – Head and Neck Surgery, McGill University, Montreal, Canada; 2 Centre for Medical Education, McGill University, Montreal, Canada; 3 Department of Emergency Medicine, McGill University, Montreal, Canada; 4 Department of Anesthesia, McGill University, Montreal, Canada; 5 Department of OTL-HNS, Montreal Children’s Hospital, 1001 Blvd. Decarie, Room A02-3015, Montreal, Canada

**Keywords:** Simulation, Otolaryngology, Pediatric airway, Residency, Education, Curriculum

## Abstract

**Background:**

A longitudinal curriculum was developed in conjunction with anesthesiologists, otolaryngologists, emergency physicians and experts in medical simulation and education.

**Methods:**

Residents participated in four different simulation-based training modules using animal models, cadavers, task trainers, and crisis scenarios using high fidelity manikins. Scenarios were based on various clinical settings (i.e. emergency room, operating room) and were followed by video-assisted structured debriefings. Participants completed both a self-assessment questionnaire and an exit survey using five-point Likert scales.

**Results:**

31 otolaryngology residents participated in the curriculum. Residents reported simulation training significantly improved technical skills such as tracheostomy, cricothyroidotomy and pediatric intubation (*p < 0.05* for all). Non-technical skills, including communication, delegation and management were significantly improved on post-test surveys in simulated crisis scenarios (*p < 0.05* for all). 90 (28/31) of participants found simulations to be very realistic. Junior residents placed increased value on didactic teaching and procedural skills, while senior residents on crisis scenarios. Survey results indicated that > 90% (28/31) of participants found the modules of the curriculum to be useful and would recommend them to others.

**Conclusion:**

A longitudinal simulation-based medical curriculum can be an effective method to teach airway management and teamwork skills to otolaryngology residents.

## Introduction

Otolaryngology–Head and Neck Surgery (OTL-HNS) is a discipline that plays an important role in the management of patients with an unstable, or potentially unstable, airway. Immediate OTL-HNS intervention is often considered lifesaving, as many patients under the care of an OTL-HNS may have failed traditional methods of securing the airway such as bag mask ventilation and intubation by other medical specialists [1].

OTL-HNS residents require the expertise to effectively and efficiently participate in airway crisis situations to ensure positive patient outcomes. However, acute or impending airway obstructions are rare events. The resulting rare and variable exposure to high-severity, low-frequency events during residency, compounded by the lack of explicit feedback based on direct observation, results in inadequate on-the-job training [2]. Therefore, it is difficult to rely on ‘on-the-job training’ for OTL-HNS residents to obtain and maintain the critical skills of airway crisis management.

Furthermore, learning and practicing these skills by novices in the acutely ill patient raises significant and important patient safety and ethical considerations. Airway crisis situations are complex, dynamic and time-sensitive contexts that pose a significant threat to patient safety, and are a major source of preventable errors [3, 4]. In the pivotal report “To Err is Human: Building a Safer Health System”, the Institute of Medicine (IOM) documented more deaths per year in the United States were attributable to preventable medical errors than to motor vehicle accidents, breast cancer or AIDS [5].

Benefits of simulation training have been well documented in graduate medical education. Other medical specialties, such as Pediatric Emergency Medicine, have reported curricula integrating simulation-based education opportunities into residency and fellowship training [6, 7]. In contrast, OTL-HNS airway workshops are typically stand-alone modules [8–11] and rely less on a systematic approach to formal curriculum development. However, stand-alone modules run in contrast to best practices in simulation-based education.

Therefore, we sought to create and implement an evidence-informed longitudinal, integrated, stage-appropriate, airway-crisis curriculum for OTL-HNS residency training, based on sound educational and simulation-based educational principles. The resultant longitudinal, four-year, simulation-based curriculum is described and evaluated.

## Methods

We conducted a prospective pilot study examining the role of a simulation-based, airway crisis management curriculum for OTL-HNS residents. Institutional ethics board approval was granted prior to the start of the study.

### Curriculum overview

The broad educational goal of the curriculum was to provide OTL-HNS residents with opportunities to learn to effectively and efficiently manage clinically relevant airway crisis situations. A multi-staged process was undertaken for the development of the longitudinal curriculum. The summary for the longitudinal curriculum, with associated stage-appropriate learning objectives can be found in Table 1. The curriculum is composed of four different modules that span the entire OTL-HNS residency program. Descriptions of the educational foundations and development of this curriculum are presented below.

**Table 1:** Curriculum Outline: Lectures, procedural skills stations and simulation scenarios for each module

Module	Target Learners	Learning Goals and Objectives	Instructional Strategies	Educational Features	Format / Dates
(1) Airway Basics	PGY level: ● 1 Disciplines: ● OTL-HNS ● All residents in Surgical Foundations (i.e. Plastics) ● Family Med ● Internal Med ● Emergency Medicine ● Oromaxillo-facial	Knowledge: Basic Approach to Airway Management	30-min lecture	Focus on acquiring knowledge and proficiency in technical skills. Clinical management on basic airway scenarios. Participants from multiple residency programs work in parallel to each other, with only limited interaction.	Format: Two half-days Development / Implementation 2008-present Data Collection 2009-present
		Procedural Skills: ● Bag-Mask Ventilation ● Endotracheal Intubation ● Alternative Airway Devices ● Basics of Tracheostomies and Trach Care ● Basics of Emergency Surgical Airway	Hands-on Practice with Task Trainers (30 min each station)		
		Clinical Management: ● Anaphylactic shock and airway edema requiring resuscitation ● Esophageal intubation with resulting hypoxemia	Simulation Scenarios with High-fidelity Manikins and Group Debriefing		
(2) Advanced Airway	PGY level: ● 2, 3 Disciplines: ● OTL-HNS ● Anesthesia ● Emergency Medicine ● General Surgery ● Sports Medicine	Knowledge: ● Management of the Difficult Airway	30-min lecture	Focus on knowledge and skills acquisition and applicability to clinical situations. Introduction of clinical management of more difficult airway scenarios. Residents from multiple programs begin to interact during the simulation scenarios.	Format: One full day Development / Implementation 2010, 2011, 2016, 2017 Data Collection 2010–2011
		Procedural Skills: ● Advanced Alternative Airway Devices ● Basics of Flexible Bronchoscopy ● Video-Assisted Intubation ● Rigid Bronchoscopy	Hands-on Practice with Task Trainers (45 min/station)		
		Procedural Skills: ● Controlled Open Tracheostomy ● Advanced Emergency Surgical Airway	Hands-on Practice with Animal Models (45 min/station)		
		Clinical Management: ● Polytrauma requiring resuscitation ● Poor oxygenation post-intubation: Mainstem intubation vs. pneumothorax	Simulation Scenarios with High-fidelity Manikins and Group Debriefing		
(3) Advanced Pediatric	PGY level: • 3, 4 Disciplines: • OTL-HNS	Knowledge: ● Crisis Resource Management ● Techniques of Open Airway Surgery	30-min lecture 10-min lectures	Emphasis on clinical management of difficult airway cases and on acquisition of CRM skills. Application of technical skills into scenarios with a team leader (within own OTL-HNS silo). CRM simulations begin with 4 subacute airway cases followed by 4 more acute cases. Roles of allied health professionals and other non-OTL physicians are scripted.	Format: Two full days Development / Implementation 2013-present Data Collection 2013, 2015, 2018
		Procedural Skills ● Rigid Bronchoscopy with Foreign Body Removal ● Flexible Bronchoscopy with Foreign Body Removal	Hands-on Practice with Task Trainers (45 min/station)		
		Procedural Skills ● Laryngotracheoplasty ● Harvesting and shaping of rib grafts ● Cricotracheal resection ● Slide tracheoplasty	Hands-on Practice with Animal Models (45 min/station)		
		Clinical Management and CRM skills ● Intraoperative management of laryngeal Papillomatosis ● Progressive Laryngeal Fracture ● Progressive Subglottic Stenosis ● Mainstem Foreign Body Aspiration ● Massive Tracheoinnominate Fistula ● Airway Fire from CO_2_ laser use ● Complete airway bbstruction from tracheal foreign body aspiration ● Acute obstruction and Ludwig’s Angina	8 Simulation Scenarios with High-fidelity Manikins, and Group Debriefing		
(4) Interdisci-plinary and Interprofessional	PGY level: ● 4, 5 Disciplines: ● OTL-HNS ● Anesthesia ● Pediatric Emergency Medicine ● Pediatric Intensive Care ● Nursing ● RT	Knowledge: ● Unique issues related to Interprofessional and Interspecialty Team Training	30 min lecture	High stress scenarios with increased case complexity. Full interprofessional and interdisciplinary team with improvisations. Shared decision making, adaptive leadership across different disciplines.	Format: Two to three half days Development / Implementation 2012-present Data Collection 2012, 2015, 2018
		Clinical Management and CRM Skills ● Trisomy 21 child with croup and possible C-spine instability ● Unanticipated difficult airway in a seizing, obese child ● Sports-related trauma with laryngeal fracture and head injury ● Excessive bleed from tracheostomy tube ● Difficult intraoperative removal of aspirated foreign body ● Massive post-tonsillectomy hemorrhage ● Anaphylaxis from intravenous antibiotic administration ● Airway fire from laser supraglottoplasty ● Acute airway obstruction from postoperative neck hematoma ● MVA polytrauma including mandibular / midface trauma	12–16 Simulation Scenarios with High-fidelity Manikins and Group Debriefing		

*ER* emergency medicine, *ICU* intensive care unit, *MVA* motor vehicle accident, *OTL* otolaryngology, *PGY* post graduate year

### Learning Objectives & Curriculum Development

Learning objectives outlined by the Royal College of Physicians and Surgeons of Canada for OTL-HNS residency training were reviewed to determine those objectives most suitable for simulation-based learning. With input from OTL-HNS residents, the four-year, longitudinal simulation-based curriculum was then iteratively developed and refined by content experts, including otolaryngologists, anesthesiologists, emergency physicians and experts in medical simulation and medical education.

We engaged in a building process where multiple conceptual frameworks informed the design and implementation of the educational curriculum. For example, Kern’s six-step approach to curriculum development [12] (problem identification, needs assessment, goals and objectives, educational strategies, implementation, evaluation and feedback) was sought to lay the groundwork. The airway curriculum was designed to be delivered in four stage-appropriate modules spanning five years, with each module having predetermined specific stage-appropriate objectives and milestones related to airway crisis management (Table 1). Modules are given no more than 6 to 12 months apart to allow for repetition and reinforcement of learned concepts.

### Simulation-based education

Different instructional strategies were purposely selected to address different learning goals and objectives. Throughout, Issenberg’s ten features of effective simulation education helped guide the educational implementation of simulation [13]. Certain educational elements that were integrated throughout the curriculum were: feedback, repetition, stage appropriate increases in task complexity, multiple learning strategies, clinical variation, defined outcomes, and representative of practice.

The instructional strategies varied, and the given strategy employed was aligned with the desired knowledge, skills (technical and non-technical) or attitudes to be acquired, and its accompanying complexity. For example, cognitive domains such as basic physiology and airway techniques were addressed by the circulation and required review of two relevant review articles, identified as mandatory pre-reading, to all trainees two weeks prior to the training sessions, and by a 30- to 60-min didactic lecture given by faculty at the start of each module.

For more psychomotor domains, experiential learning using simulation was commonly adopted throughout the curriculum. Technical skills training relied on skill-based stations with the following format: demonstration by content experts, supervised hands-on practice with task trainers and/or cadaveric animal models, followed by immediate individualized feedback from faculty members.

### Scenario development and debriefing

Simulated scenarios focused on crisis resource management (CRM) skills, and interprofessional / interdisciplinary team training. Scenario design and debriefing sessions were based on Salas’ concepts underlying successful teamwork; highlighting key competencies of team leadership, mutual performance monitoring, backup behavior, adaptability, team orientation, shared mental models, mutual trust and closed-loop communication [14]. In addition, the core concepts provided by D’Amour et al. in their sentinel review on interprofessional collaboration (fundamental concepts of sharing, partnership, power, interdependency and process) were added to the advanced modules dealing with complex management airway crisis [15].

Residents both participated in and observed simulated scenarios on high-fidelity mannequins. Each scenario lasted 10 to 12 min, and took place in different yet clinically relevant environments such as the operating room, emergency room and post-anesthetic care unit. Scenarios were designed to incorporate specific cognitive, affective and psychomotor objectives being developed in conjunction with simulation and content experts, and underwent simulator programming, piloting, revision and modification to ensure realism and quality.

Each scenario was followed by a 20-min structured debriefing session where trainees were encouraged to reflect on their reasoning, decision-making, and actions. Instructors could identify and playback brief video clips of critical moments during the scenario for further in-depth discussion.

### Study setting and participants

All courses took place either at McGill University’s Arnold and Blema Steinberg Medical Simulation Centre, the Montreal Children’s Hospital, both in Montreal, Quebec, Canada or at Western University’s London Health Science Centre in London, Ontario, Canada. No formal exclusion criteria were used in order to maximize resident availability, and participants represent a cohort convenience sample. All participants were excused from clinical duties to attend the course, and refusing to participate in the evaluation of the curriculum did not preclude participation in the airway curriculum. Ethics approval was obtained from McGill University.

### Survey

After each module, participants completed a self-assessment questionnaire on their self-perceived improvement in knowledge, technical and non-technical CRM skills gained. Individual and team CRM skills inluded in the self-assessment are listed in Table 2. Residents also completed an exit survey addressing perceived usefulness of the program and an overall program evaluation. The exit survey included several open-ended questions eliciting feedback regarding strengths and weaknesses of the courses in order to facilitate continued educational quality improvement. All questions were based on a 5-point Likert scale (where 1 = “strongly disagree” or “poor”, and 5 = “strongly agree” or “expert” depending on the question). Pre and post survey results were compared using the independent student t-test. Descriptive statistics were used to analyze the quantitative portion of the survey, while content analysis was applied to qualitative responses.

**Table 2:** Individual and team CRM skills assessed

Individual CRM Skills	1) Identify and formulate a plan for an airway crisis 2) Effective leader in an airway crisis 3) Ensure effective communication 4) Delegate tasks 5) Use all available resources 6) Maintain a global perspective (avoid fixation errors) 7) Airway knowledge 8) Technical skills 9) Recognition of own limitations
Team CRM Skills	1) Identify issues in a crisis situation that could be better handled by others (call for help) 2) Develop a common plan to manage an airway crisis 3) Collaborate with others to anticipate and plan for issues in a crisis.

Adapted from Stanford Medicine Emergency Manual for crisis resource management key skills [31]

## Results

### Curriculum

For a full description of the modules, content, target participants, educational objectives and instructional modalities, please refer to Table 1. Modules 1 and 2 focus on knowledge acquisition and technical skills proficiency, and introduce learners to less complex airway simulation scenarios. Here, the OTL-HNS residents learn alongside residents from other disciplines with limited team interaction. Instead all participants are exposed to the same educational material as equal, but relatively non-interactive learners. Data from a subset of Module 2 has been presented in a study by our group [16].

The more advanced Module 3, while reinforcing advanced technical skills, largely concentrates on the introduction of basic concepts of the non-technical skills of crisis resource management (CRM), such as leadership and followership in teams. In this module, nurses and respiratory therapists provide assistance by acting as confederates and playing their respective scripted roles in the interprofessional team in order to increase the complexity and realism of the simulated scenarios. Thus, the OTL-HNS residents apply their technical skills in more advanced, acute team situations. Data from a subset of this module has been presented elsewhere by members of our group [17].

Module 4 incorporates and consolidates all aspects into an interprofessional and interdisciplinary setting to best reflect clinical reality. Senior residents and fellows from Emergency Medicine and Anesthesia residency programs fully participate in these simulations, many of whom are in-situ (occurring in the actual clinical environment, and not at the simulation lab). Thus, through exposure of 16 different complex airway scenarios within this module, the OTL-HNS residents learn complex skills such as shared-decision making, and are introduced to the role of systems-based issues in patient safety.

### Survey

**Fig. 1:**
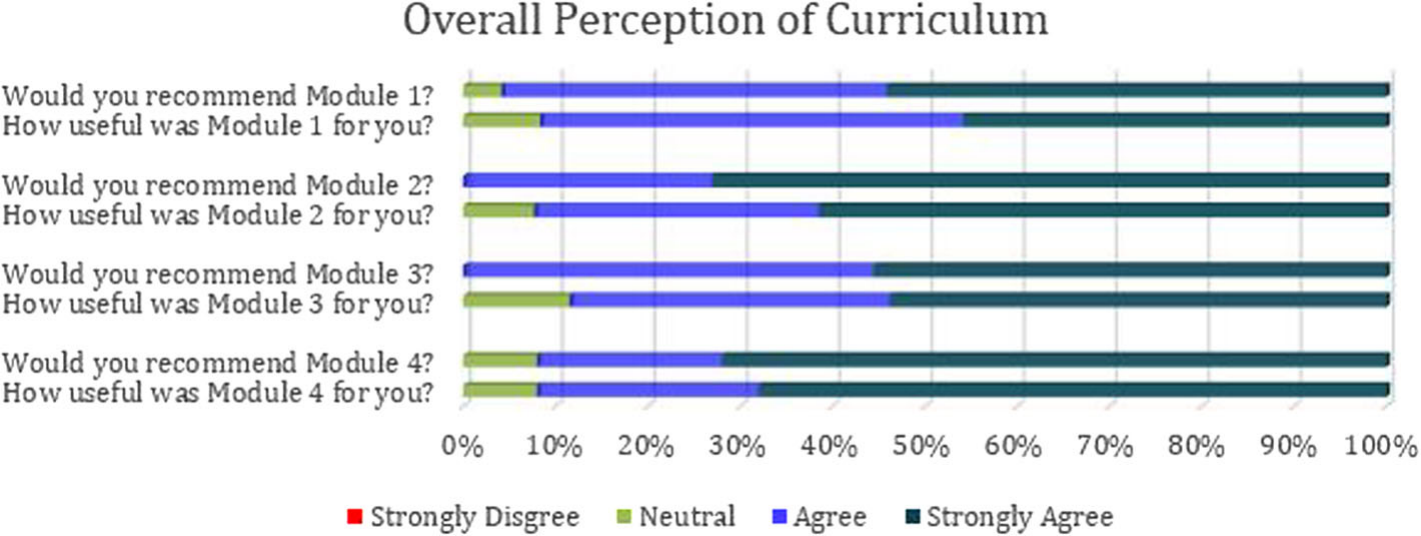
Summary of responses to exit surveys administered after each module. All findings were statistically significant with *p* < 0.05

In total, 31 OTL-HNS residents have participated in the curriculum, with various modules being implemented since 2008. When taking into account residents from other programs, Modules 1, 2, 3 and 4 had a total of 480, 48, 31 and 125 participants, respectively. Of note, due to the complexity of the curriculum, module implementation occurred non-sequentially, and data collection did not occur every year. Figure 1 summarizes the overall satisfaction of participants across the modules. More than 90% (28/31) of participants found the modules were useful and would recommend the course to others.

**Fig. 2:**
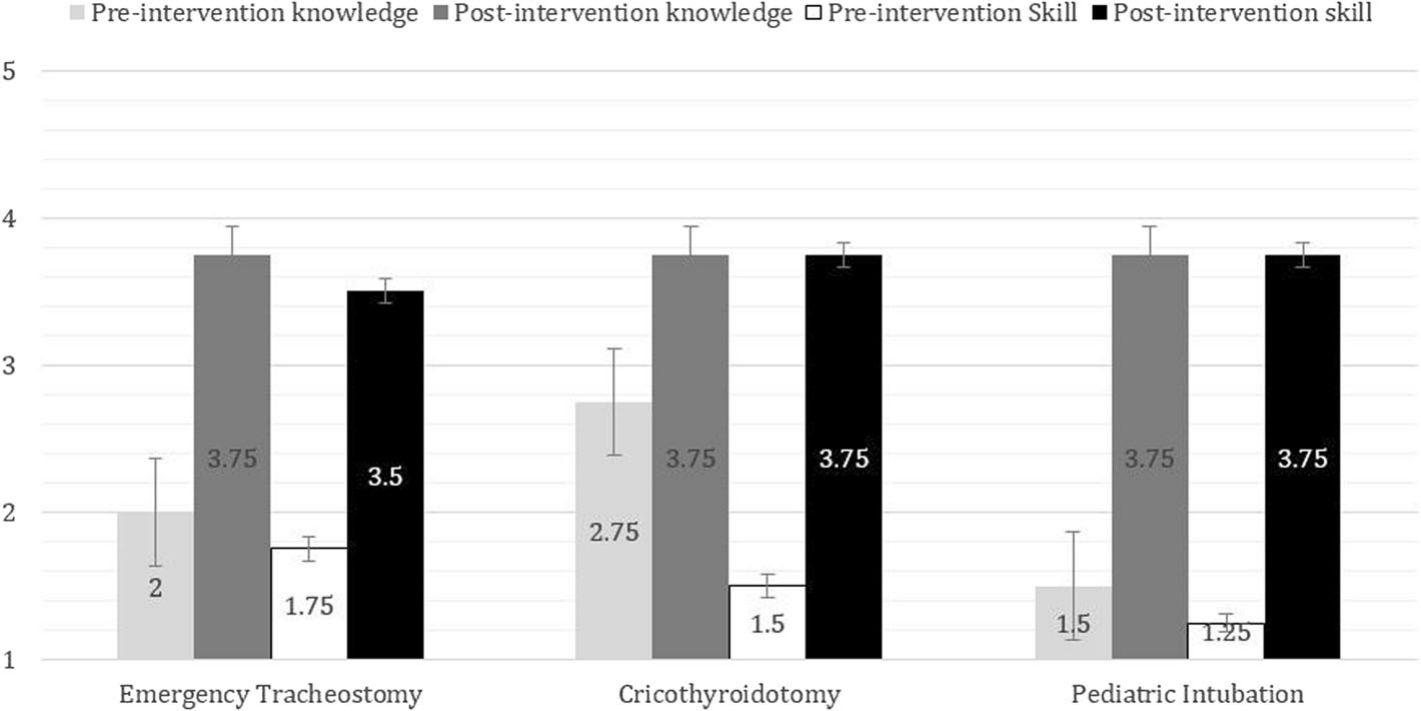
Module 1 comparision of self-perceived assessment of *knowledge* and *skill* before and after the intervention. Scores were rated on a Likert scale of 1–5 (1 = Poor, 5 = Expert). All findings were statistically significant with *p* < 0.05

For Modules 1 and 2, residents reported that simulation training significantly improved their level of knowledge and technical skills prior to and after undergoing simulation for tracheostomy, cricothyroidotomy and pediatric intubation (Fig. 2).

**Fig. 3:**
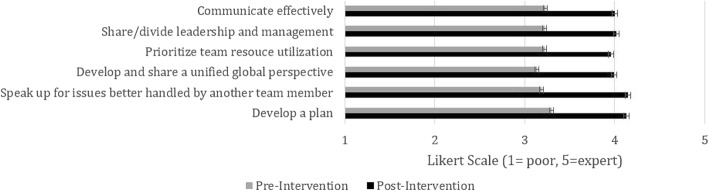
Module 4 comparision of self-reported assessment of *Non-Technical Skills* before and after the intervention. Scores were rated on a Likert scale of 1–5 (1 = Poor, 5 = Expert. All items were statistically significant with a *p* < 0.05. CRM: Crisis resource management. RT: Respiratory therapist

An overall significant self-reported increase in non-technical CRM skills in Modules 3 and 4 was reported when comparing pre and post course surveys (mean increase of pre and post course Likert score of 0.7 (±0.05 SE), Fig. 3). CRM simulations were rated as very realistic according to participants (mean of 4.3 on 5; Likert scale of 1 (poor) to 5 (excellent)). 84% of participants reported that they felt that the simulation modules should be a mandatory part of their residency training. Junior residents placed increased value on didactic teaching and procedural skills, while more senior residents placed more value on the educational components centered on CRM skill development.

## Discussion

Nationwide surveys in the United Kingdom (UK) and Canada have explored the perceived level of competency in management of airway emergencies amongst OTL-HNS residents [17, 18]. Both studies found that despite mandatory advanced cardiac life support training, nearly half of all OTL-HNS residents lack confidence in their airway skills [17, 18]. In Canada, residents report mean comfort levels of 2.8 (on a 5-point Likert scale) for pediatric airway management, corresponding at best to the anchor of “neutral” [18]. Barriers to learning difficult airway management include infrequent training opportunities, early staff intervention, and societal pressures for increased patient safety. In an attempt to remedy this training gap, we developed a four-module, simulation-based curriculum for pediatric airway crisis management designed specifically for OTL-HNS residents and integrated longitudinally in our training program. This is the first study, to our knowledge, that describes a longitudinal airway curriculum for OTL-HNS residents purposefully grounded in educational principles and best practices. A high overall satisfaction from participants and evidence of knowledge transfer in technical and non-technical CRM skills was achieved.

The reported simulation-based curriculum proposes a graduated approach to training of OTL-HNS residents in adult and pediatric airway crisis. Each educational module has a unique and stage-appropriate set of specifically defined objectives, and attainment of these objectives is a prerequisite for participation in subsequent modules. This essential feature of the curriculum design allows for core concepts to build upon the foundations from the previous modules, using increasingly participatory instructional strategies, and facilitates incorporating increasing complexity across simulated scenarios and curricular components.

Moreover, our curriculum design appears to be well accepted and appreciated by the residents. Far from a static product, this curriculum is a dynamic educational process, and we engage on continual educational quality improvement based on instructor debriefings, participant feedback, and close monitoring of literature reporting on educational and airway management best-practices. As a specific example, Module 1 was adapted to target multiple medical disciplines after comments about generalizing the material to other trainees was voiced. CRM modules included non-medical staff such as nurses and respiratory therapists after feedback from residents. Survey responses from residents continue to be collected across the different modules to guide module changes. The survey results support a Kirkpatrick level 1, with residents showing great satisfaction in the modules. Results from the advanced pediatric course (Module 3) have shown evidence of learning and retention, corresponding to a Kirkpatrick level 2 [17]. Further, some work has documented a positive relationship between resident self-assessment surveys and resident performance during simulated resuscitations [19, 20]. In addition, the airway curriculum addresses many of the Entrusted Professional Activities (EPAs) required by the Competence-Based Medical Education (CBME) framework put forth by the Royal College of Physicians and Surgeons of Canada. Although designed prior to the recent introduction of CBME, the airway curriculum covers (either partially or in full) over six EPAs found throughout the course of residency. Examples include Foundation EPA 1 (*“providing initial clinical assessment, investigation and development of a management plan for patients with acute upper airway obstruction”)*, and Core EPA 5 (*“providing emergency surgical management for patients with acute airway problems”)*.

With growing evidence supporting the value of simulation-based training in improving technical skills, it is becoming a mainstay of surgical training. In their recent review, Zendelas et al. found that simulation, when compared to traditional instructional strategies, resulted in reduced surgical time and higher performance ratings for laparoscopic surgery [21]. More importantly, simulation-based education has translated into improved patient outcomes in multiple clinical settings including technical skills and crisis situations [22]. Simulation-based airway courses in OTL-HNS have been described by various authors [8, 10, 23–25]. However, these programs focus more on individual knowledge and technical skills, and may inadequately address the non-technical skills needed for individuals working together to quickly, collaboratively, and competently manage a crisis. Our findings suggest that junior residents placed relatively higher value on didactic teaching and hands-on procedural skills stations, suggesting the need to fill the more basic knowledge and technical skill gaps outside of high-fidelity simulation scenarios. In contrast, senior residents reported valuing the more complex crisis simulation scenarios focusing more on the non-technical CRM skills of communication, leadership and team training; reinforcing the need for stage- and task-appropriate educational objectives, instructional approaches, and a longitudinal structured curriculum.

Crisis resource management, focuses on non-technical skills necessary for effective team process; including skills such as leadership, role allocation, decision-making and situational awareness [26]. Indeed, poor CRM skills have been found to account for 70–80% of medical errors in crisis situations [19, 27–30]. Airway courses by Zirkle et al. [8] and specifically by Volk et al. [9] have brought CRM to the forefront of the OTL-HNS literature. However, to date, there is no study that has described an airway curriculum where OTL-HNS residents progress from one module to another in a structured stage-appropriate fashion upon completion of predetermined milestones.

Our study has several limitations. We did not compare our curriculum to a control group where residents underwent traditional non-simulation teaching methods, as we felt that denying resident participation would have a detrimental effect on learning and performance. Finally, we are unable to comment on the generalizability of our educational curriculum beyond our institution, although modules with similar educational objectives and instructional approaches are currently being implemented at other university centers. Module 2 underwent a hiatus as the lead instructor was unable to run the entirety of the modules at all times. The ideal curriculum would have all four modules concurrently to address all levels of OTL-HNS residents. Lastly, an over-encompassing evaluation does not exist at this point to evaluate the entirety of the curriculum.

## Conclusion

A simulation-based curriculum can be an effective method to teach airway crisis management and teamwork skills to OTL-HNS residents in a purposeful, stage-appropriate approach. Residents rated the modules within the curriculum as a positive learning experience and reported improvements in knowledge, technical and non-technical skills.
